# Inhibitor of DNA Binding 1 Is Induced during Kidney Ischemia-Reperfusion and Is Critical for the Induction of Hypoxia-Inducible Factor-1*α*

**DOI:** 10.1155/2016/4634386

**Published:** 2016-04-05

**Authors:** Dan Wen, Yan-Fang Zou, Yao-Hui Gao, Qian Zhao, Yin-Yin Xie, Ping-Yan Shen, Yao-Wen Xu, Jing Xu, Yong-Xi Chen, Xiao-Bei Feng, Hao Shi, Wen Zhang

**Affiliations:** ^1^Department of Nephrology, Ruijin Hospital, School of Medicine, Shanghai Jiaotong University, Shanghai 200025, China; ^2^Department of Science and Education, MinHang Central Hospital, Shanghai, China; ^3^Shanghai Universities E-Institute for Chemical Biology, Key Laboratory of Cell Differentiation and Apoptosis of National Ministry of Education, School of Medicine, Shanghai Jiaotong University, Shanghai 200025, China; ^4^Shanghai Institute of Hematology, Shanghai, China

## Abstract

In this study, rat models of acute kidney injury (AKI) induced by renal ischemia-reperfusion (I/R) and HK-2 cell models of hypoxia-reoxygenation (H/R) were established to investigate the expression of inhibitor of DNA binding 1 (ID1) in AKI, and the regulation relationship between ID1 and hypoxia-inducible factor 1 alpha (HIF-1*α*). Through western blot, quantitative real-time PCR, immunohistochemistry, and other experiment methods, the induction of ID1 after renal I/R in vivo was observed, which was expressed mainly in renal tubular epithelial cells (TECs). ID1 expression was upregulated in in vitro H/R models at both the protein and mRNA levels. Via RNAi, it was found that ID1 induction was inhibited with silencing of HIF-1*α*. Moreover, the suppression of ID1 mRNA expression could lead to decreased expression and transcription of HIF-1*α* during hypoxia and reoxygenation. In addition, it was demonstrated that both ID1 and HIF-1*α* can regulate the transcription of twist. This study demonstrated that ID1 is induced in renal TECs during I/R and can regulate the transcription and expression of HIF-1*α*.

## 1. Introduction

Ischemia-reperfusion- (I/R-) induced acute kidney injury (AKI) is a common clinical event and frequently results in the development of chronic kidney disease (CKD) or end-stage renal disease, leading to high mortality [1]. Tubular epithelial cells (TECs) are vulnerable to renal I/R injury, contributing significantly to the loss of kidney function [2]. Following I/R injury to the kidneys, the TECs, which have a relatively high oxygen requirement, are easily injured, because hypoxia can trigger a series of reactions and lead to cell apoptosis and/or necrosis and tissue damage. However, renal TECs have the potential to regenerate after ischemia. The normal repair process after TECs injury is critical for the recovery of kidney function and structure.

Hypoxia-inducible factor-1 alpha (HIF-1*α*) is a master gene switch for major adaptive responses to hypoxia. It is a basic helix-loop-helix (bHLH) transcription factor that is degraded during normoxia. During hypoxia, HIF-1*α* accumulates and dimerizes with constitutively expressed HIF-1*β* and mediates the expression of target genes, including erythropoietin and vascular endothelial growth factor [3]. Many studies have demonstrated that HIF-1*α* is activated in AKI with or without ischemia and serves to ameliorate AKI by improving hypoxia [4]. Thus, HIF-1*α* is critical for the survival of TECs [5]. As the normal expression of HIF-1*α* plays an important role in the survival of TECs, regulators of HIF-1*α* at the transcriptional or protein level might have potential applications. Kim et al. found that inhibitor of DNA binding 1 (ID1) can enhance the stability and activity of HIF-1*α* in human endothelial and breast cancer cells [6]. Whether ID1 can regulate HIF-1*α* in TECs has not been reported, and the relationship between ID1 and HIF-1*α* remains to be completely understood.

ID1 is also a bHLH transcription factor and is referred to as an inhibitor of DNA binding because it does not possess a basic DNA-binding domain. The ID family functions as dominant-negative regulators of other bHLH proteins through the formation of inactive heterodimers with intact bHLH transcription factors [7]. Overexpression of ID1 is associated with cell dedifferentiation and proliferation in several cell lineages [8]. It has been shown that the expression of ID1 is increased in CKD models [9]. However, the role of ID1 in kidney I/R injury is not clear, and whether hypoxia can upregulate ID1 in TECs remains to be explored.

Based on the possibility of ID1 induction in AKI and the potential relationship between ID1 and HIF-1*α*, the authors hypothesized that ID1 is an important factor in AKI that may be induced under hypoxia and may regulate the expression of HIF-1*α* in TECs. In the present study, the expression of ID1 in both in vitro and in vivo kidney I/R models was evaluated and its effects on HIF-1*α* expression in HK-2 cells were analyzed. It was observed that ID1 expression was upregulated during I/R and hypoxia-reoxygenation (H/R) and that ID1 can regulate HIF-1*α* expression at the transcriptional level.

## 2. Materials and Methods

### 2.1. Rat Kidney I/R Injury Model

Male Sprague-Dawley rats weighing 250–280 g were obtained from the Animal Center of Ruijin Hospital. The experimental protocol was approved by the Ethics Committee for Animal Care and Use of the Research Center for Experimental Medicine of Ruijin Hospital. These rats were divided into groups of 5-6 animals for each condition. Renal I/R injury was applied under inhalational anesthetics by clamping bilateral renal pedicles for 45 min, and the animals were kept on a warming table at 37°C during the surgery. Rats in the sham group were operated using the same procedure without clamping. Rats were sacrificed at different time points before ischemia and during reperfusion, and their blood and kidney cortex specimens were obtained for analysis.

### 2.2. Renal Function Test

Serum creatinine and blood urea nitrogen, recognized as renal function indexes, were measured in serum samples collected from rats. The detection methods for the 2 parameters were described previously [10].

### 2.3. Cell Culture and H/R Model

Human proximal tubular cells (HK-2, CRL-2190) were purchased from ATCC. HK-2 cells were cultured in Dulbecco's Modified Eagle's Medium (DMEM/F12 medium; GIBCO) containing 10% fetal bovine serum (FBS; GIBCO) in a humidified atmosphere with 5% CO_2_ at 37°C. The medium was changed every 2 days, and all experiments were performed using cells in a 70%–90% confluent monolayer. For exposure to hypoxia, new complete medium was added to cell monolayers after 24 hours of serum starvation. Culture plates were placed in a humidified hypoxic chamber (Thermo Electron) with an atmosphere of 1% O_2_, 94% N_2_, and 5% CO_2_ for 24 hours. After exposure to hypoxia, the medium was refreshed again and the plates were moved to a normoxic cell incubator (21% O_2_ and 5% CO_2_).

### 2.4. Immunohistochemistry

Parts of kidney cortex specimens were fixed in 10% neutral buffered formalin, and 4 *μ*m paraffin-embedded kidney sections were treated with peroxidase blocking solution and goat serum (Fuzhou Maixin, China) at room temperature. The sections were incubated with primary antibodies (ID1; Santa Cruz Biotechnology, HIF-1*α*; Santa Cruz Biotechnology) overnight at 4°C and then with biotinylated anti-IgG secondary antibody (Fuzhou Maixin, China). Streptavidin conjugated with horseradish peroxidase (HRP) was used to identify biotin, which was visualized by staining with DAB solution (Fuzhou Maixin, China). Sections were imaged with a spot-cam digital camera (Carl Zeiss).

### 2.5. Western Blot Analysis

Kidney cortex tissue that had been stored at −80°C was homogenized in radioimmunoprecipitation (RIPA) buffer (Sigma-Aldrich) containing phenylmethylsulfonyl fluoride for 30 min on ice followed by centrifugation (12,000 rpm, 20 min) at 4°C and collection of supernatants. HK-2 cells were washed with phosphate-buffered saline (PBS) and lysed in cell lysis buffer containing 4% sodium dodecyl sulfate, 20% glycerol, 100 mM dithiothreitol, and Tris-HCl, pH 6.8. The cell lysates were also centrifuged (12,000 rpm, 10 min) at 4°C. Then, 40 *μ*g of supernatant proteins was loaded and separated by 8%–15% sodium dodecyl sulfate-polyacrylamide gel electrophoresis. Proteins were transferred to a polyvinylidene difluoride membrane (Millipore) after electrophoresis. The blot was blocked with 5% dried skim milk in Tris-buffered saline containing 0.1% Tween-20 (TBST) at room temperature for 2 hours, followed by incubation with primary antibodies overnight at 4°C. Dilutions of 1 : 2000 for *β*-actin (Santa Cruz Biotechnology), 1 : 1000 for HIF-1*α* (BD Transduction Laboratories, Abclonal), 1 : 200 for ID1 (Santa Cruz Biotechnology), 1 : 1000 for vimentin (Santa Cruz Biotechnology), and 1 : 200 for twist (Santa Cruz Biotechnology) were used. The membranes were washed and then incubated with HRP-conjugated secondary antibodies (Cell Signaling Technology) at room temperature for 2 hours. After several washes, the proteins were visualized with enhanced chemiluminescence (ECL) kits (Amersham) and quantified by gray scale analysis by using ImageJ software (National Institutes of Health). For all western blots, *β*-actin was used as an internal control.

### 2.6. Quantitative Real-Time PCR

Total RNA was extracted from HK-2 cells using TRIzol reagent (Invitrogen). Reverse transcription for cDNA synthesis was performed with a High Capacity cDNA Reverse Transcription Kit (Invitrogen). Expression levels of ID1, HIF-1*α*, vimentin, twist, and *β*-actin were quantified by real-time PCR using SYBR Premix Ex Taq (TaKaRa, Dalian, China) according to the manufacturer's cycling protocol, and amplification was performed on triplicate samples. The PCR primers used were as follows: ID1 sense (5′-CTACGACATGAACGGCTGTTACTC-3′) and ID1 antisense (5′-CTTGCTCACCTTGCGGTTCT-3′), HIF-1*α* sense (5′-GTTTACTAAAGGACAAGTCACC-3′) and HIF-1*α* antisense (5′-TTCTGTTTGTTGAAGGGAG-3′), vimentin sense (5′-CCAGAGGGAGTGAATCCAGA-3′) and vimentin antisense (5′-AGATGGCCCTTGACATTGAG-3′), twist sense (5′-GCAAGATTCAGACCCTCAAG-3′) and twist antisense (5′-CATCCTCCAGACCGAGAAG-3′), and *β*-actin sense (5′-GATCTTCGGCACCCAGCACAATGAAGATC-3′) and *β*-actin antisense (5′-AAGTCATAGTCCGCCTAGAAGCAT-3′). Gene expression was expressed as 2^−ΔΔ(Ct)^, and the control samples were unstimulated cells.

### 2.7. Small Interfering RNA Transfection in HK-2 Cells

The small interfering RNA (siRNA) technique was used for gene silencing. SiRNA for ID1 and HIF-1*α* and a negative control were designed and purchased from Invitrogen (ID1-siRNA-427, ID1-siRNA-269, ID1-siRNA-529; HIF-1*α*-siRNA-1511, HIF-1*α*-siRNA-964, and HIF-1*α*-siRNA-2107). HK-2 cells were cultured in 6-well plates, and complete medium was removed at 50%–60% confluence when Opti-MEM (Invitrogen) was added. ID1 or HIF-1*α* siRNA and negative siRNA were diluted in Opti-MEM and incubated with diluted Lipofectamine RNAiMAX Reagent (Invitrogen) for 5 min according to the manufacturer's protocol. SiRNA-lipid complexes were added to cells and 25 pmol siRNA was used per well. After 24 hours, the medium was replaced by DMEM/F12 medium containing 10% FBS, and the plates were moved to the hypoxic chamber for application of the H/R procedure as described above. Western blot and real-time PCR analyses were used to confirm the transfection efficiency.

### 2.8. Statistical Analysis

All data were expressed as mean ± standard deviation (SD), and the results for 2 groups were compared by *t* tests. Multigroup comparisons were made using one-way analysis of variance (ANOVA). Probability values of <.05 were considered to be statistically significant. All statistical analyses were performed with SPSS software (Version 11.0; SPSS, Inc.).

## 3. Results

### 3.1. ID1 and HIF-1*α* Expressions Are Induced after Renal I/R In Vivo

**Figure 1:**
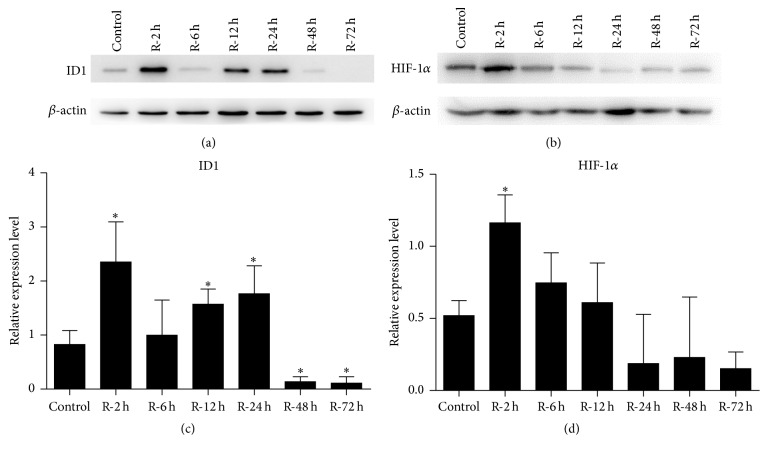
ID1 and HIF-1*α* protein induction in the rat I/R injury model. (a, b) Western blot analysis of ID1 and HIF-1*α* expression in the kidney cortex after I/R. The expression of *β*-actin was examined as the loading control. A representative blot from 3 independent experiments is shown. (c, d) The histogram shows the average volume densities corrected for *β*-actin (*n* = 3). ^*∗*^ *P* < .05 compared with the preischemia controls. ID1, inhibitor of DNA binding 1; HIF-1*α*, hypoxia-inducible factor-1 alpha; I/R, ischemia-reperfusion.

To investigate ID1 and HIF-1*α* expression in vivo, the I/R model in Sprague-Dawley rats was established as described previously [10]. Western blot analysis of kidney cortex showed the induction of ID1 and HIF-1*α* expression during reperfusion in vivo. ID1 expression was elevated immediately and transiently after reperfusion for 2 hours, and a reinduction was observed after reperfusion for 12 hours (Figure 1(a)). After statistical analysis of the grey level, ID1 was determined to be induced significantly after reperfusion for 2, 12, and 24 hours, and it was markedly reduced after 48 and 72 hours of reperfusion (Figure 1(c)). HIF-1*α* protein was elevated after reperfusion for 2 hours (Figure 1(b)), and the analysis of the grey level showed significant upregulation of it (Figure 1(d)).

**Figure 2:**
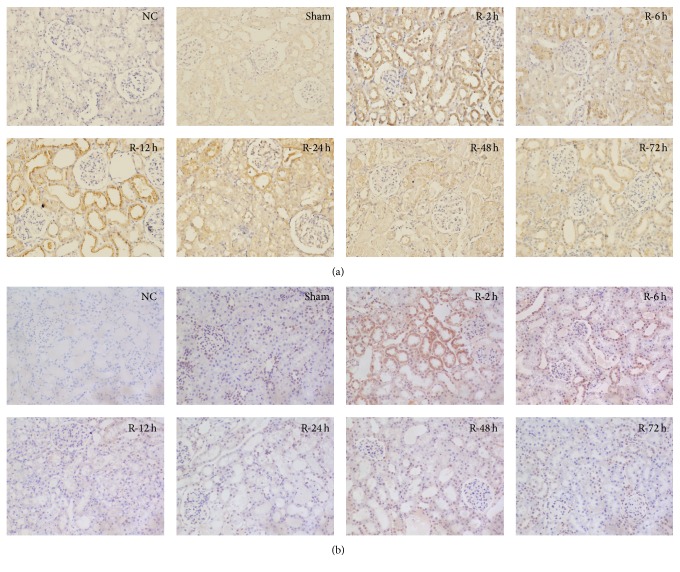
Location of ID1 and HIF-1*α* expression in I/R-injured rat kidney tissue. Immunohistochemical staining for ID1 and HIF-1*α* in the kidney tissue of I/R and sham-operated rats. (a) Increased staining for ID1 in renal TECs after 2, 6, 12, and 24 hours of reperfusion. (b) Increased staining for HIF-1*α* in renal TECs after 2 hours of reperfusion. Original magnification, ×200. ID1, inhibitor of DNA binding 1; HIF-1*α*, hypoxia-inducible factor-1 alpha; I/R, ischemia-reperfusion; TECs, tubular epithelial cells.

Immunohistochemical staining was applied to determine the location of ID1 and HIF-1*α* expression. As expected, ID1 was mainly expressed in the cytoplasm of renal TECs and was strongly expressed after reperfusion for 2 and 12 hours (Figure 2(a)). HIF-1*α* was mainly expressed in TECs, in both the cytoplasm and the nucleus. The elevated expression of HIF-1*α* was observed after reperfusion for 2 hours (Figure 2(b)).

### 3.2. Establishment of H/R Model in HK-2 Cells

To study ID1 expression in vitro, an H/R model in HK-2 cells was established, which reproduces the renal I/R injury process to a great extent. Because there is no standard or classical H/R model, a preliminary study was conducted, and the authors found that oxygen deprivation for 24 hours may mimic in vivo I/R. The expression of HIF-1*α* and dedifferentiation-related markers were the main indicators for determining the establishment of the H/R model in this study.

**Figure 3:**
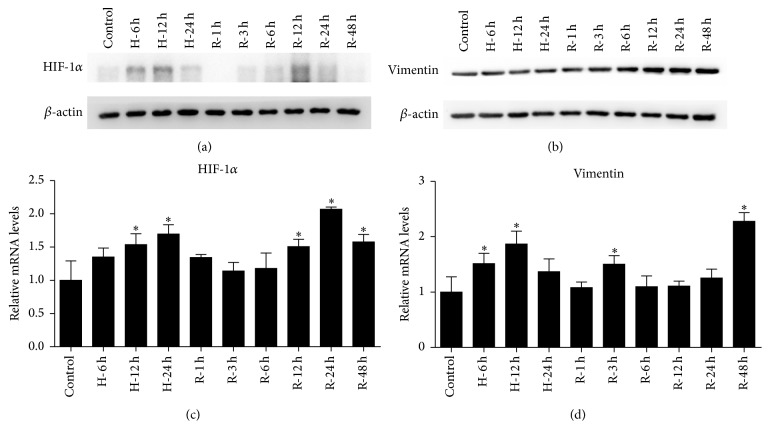
HIF-1*α* and vimentin induction in the HK-2 cell H/R model. (a, b) Western blot analysis of HIF-1*α* and vimentin in HK-2 cells under hypoxia (H) and reoxygenation (R). Cells for reoxygenation experienced 24 hours of hypoxia. *β*-actin was examined as the loading control. A representative blot from 3 independent experiments is shown. (c, d) Quantitative RT-PCR was used for the analysis of HIF-1*α* and vimentin mRNA levels in HK-2 cells under H/R. Data are expressed as mean ± SD for HIF-1*α* and vimentin levels using *β*-actin mRNA as an internal control (*n* = 3). ^*∗*^ *P* < .05 compared with the prehypoxia controls. HIF-1*α*, hypoxia-inducible factor-1 alpha; H/R, hypoxia-reoxygenation.

The detailed dynamic expression of HIF-1*α* and vimentin by western blot (Figures 3(a) and 3(b)) was observed. HIF-1*α* expression was induced obviously during hypoxia and reduced immediately at the beginning of reoxygenation, followed by a transient induction during reoxygenation. Vimentin expression was increased gradually and significantly upregulated during the reoxygenation phase. qRT-PCR was performed to estimate the mRNA levels of HIF-1*α* and vimentin (Figures 3(c) and 3(d)). The HIF-1*α* mRNA levels were significantly changed during the latter half of hypoxia and reoxygenation. In addition, there was an upregulation of vimentin at the transcription level.

These results suggest that HIF-1*α* is induced during both hypoxia and reoxygenation and that HIF-1*α* is regulated more sensitively and earlier at the protein level than at the transcriptional level. The upregulation of vimentin indicates a dedifferentiation process in this model.

### 3.3. ID1 Expression Is Induced during H/R and Is Regulated by HIF-1*α*

**Figure 4:**
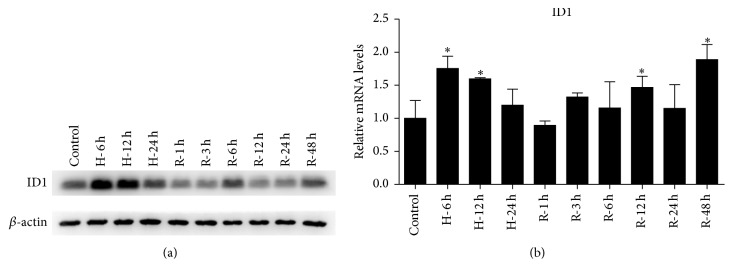
ID1 induction in HK-2 cells H/R model. (a) Western blot analysis of ID1 in HK-2 cells under hypoxia (H) and reoxygenation (R). Cells for reoxygenation experienced 24 hours of hypoxia. *β*-actin served as the loading control. A representative blot from 3 independent experiments is shown. (b) Quantitative RT-PCR was used for analysis of ID1 mRNA levels in HK-2 cells under H/R. Data are expressed as mean ± SD for ID1 levels. *β*-actin mRNA served as the internal control (*n* = 3). ^*∗*^ *P* < .05 compared with the prehypoxia controls. ID1, inhibitor of DNA binding 1; H/R, hypoxia-reoxygenation.

After establishment of the H/R model in HK-2 cells, ID1 expression was assessed through western blot and qRT-PCR. The expression of ID1 was significantly increased during hypoxia and reduced following reoxygenation (Figure 4(a)). After 6 and 48 hours of reoxygenation, ID1 was upregulated again, although not remarkably. Moreover, the mRNA levels of ID1 were markedly elevated during hypoxia and reinduced after reoxygenation (Figure 4(b)).

**Figure 5:**
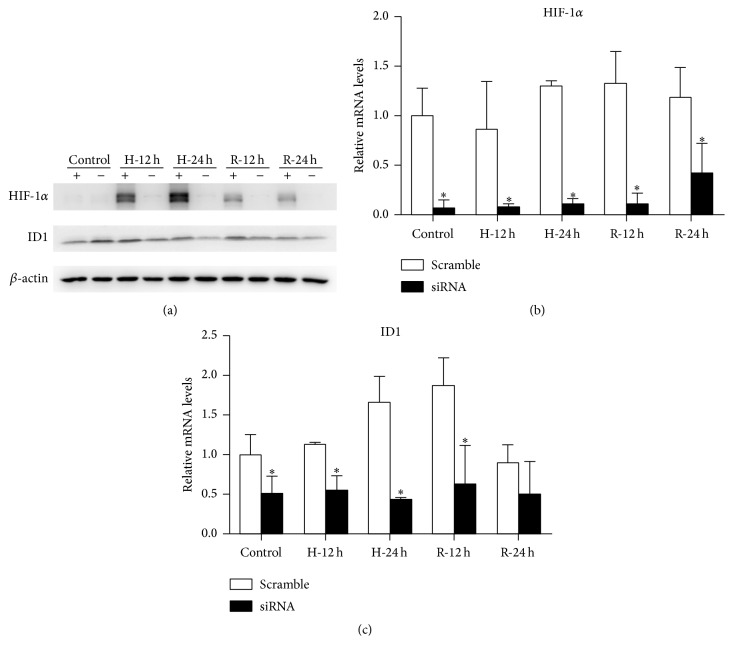
HIF-1*α* is required for ID1 induction in the H/R model. HK-2 cells were transiently transfected with specific siRNA for HIF-1*α* or siRNA for negative control and then subjected to H/R. (a) In cells transfected with HIF-1*α* siRNA, the expression of HIF-1*α* was markedly inhibited and ID1 was suppressed, although not remarkable. *β*-actin served as the loading control. A representative blot from 3 independent experiments is shown. (b, c) In cells transfected with HIF-1*α* siRNA, relative mRNA levels of HIF-1*α* were efficiently reduced. Relative mRNA levels of ID1 were decreased significantly at the prehypoxia, 12-hour and 24-hour time points for hypoxia, and after 12 hours of reoxygenation. *β*-actin mRNA was used as the internal control (*n* = 3). ^*∗*^ *P* < .05 compared with the negative control (scramble). ID1, inhibitor of DNA binding 1; HIF-1*α*, hypoxia-inducible factor-1 alpha; H/R, hypoxia-reoxygenation.

Subsequently, siRNAs for HIF-1*α* were used to investigate whether ID1 was mediated by HIF-1*α*. The expression of HIF-1*α* was inhibited and this outcome was confirmed by western blot and qRT-PCR (Figures 5(a) and 5(b)). Cells in which HIF-1*α* expression was suppressed showed reduced expression of ID1 through H/R, and mRNA levels were significantly downregulated (Figures 5(a) and 5(c)). The results indicate that ID1 is induced in the H/R model and is regulated by HIF-1*α*.

### 3.4. ID1 Regulates the Induction of HIF-1*α* Expression in Response to H/R In Vitro

**Figure 6:**
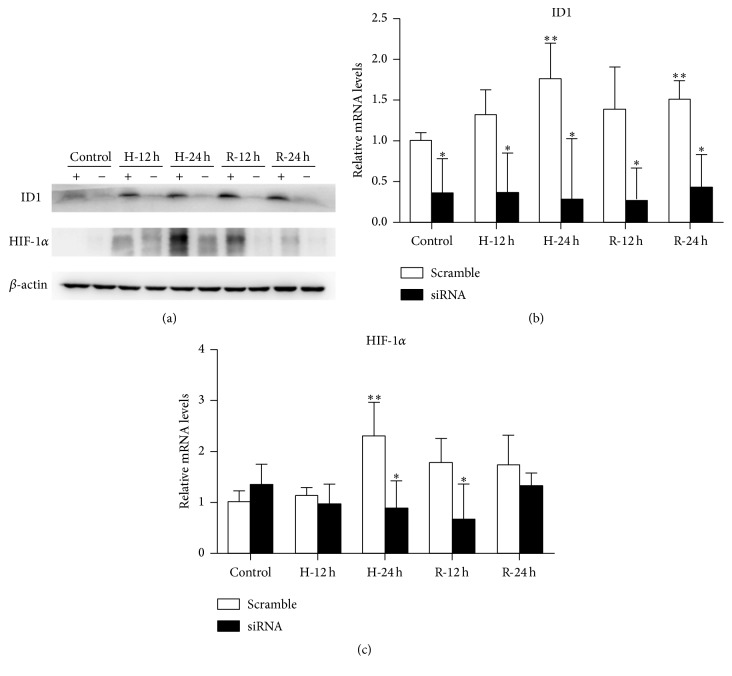
ID1 is required for HIF-1*α* induction in the H/R model. HK-2 cells were transiently transfected with specific siRNA for ID1 or siRNA for negative control and then subjected to H/R. (a) In cells transfected with ID1 siRNA, the expression of ID1 was markedly inhibited. HIF-1*α* protein expression was remarkably suppressed after exposure to hypoxia for 24 hours or to reoxygenation for 12 or 24 hours. *β*-actin expression was examined as the loading control. A representative blot from 3 independent experiments is shown. (b, c) In cells transfected with ID1 siRNA, relative mRNA levels of ID1 were efficiently reduced. Relative mRNA levels of HIF-1*α* were decreased significantly after 24 hours of hypoxia and 12 hours of reoxygenation. *β*-actin mRNA served as an internal control (*n* = 3). ^*∗*^ *P* < .05 compared with the negative control (scramble). ^*∗∗*^ *P* < .05 compared with the prehypoxia controls. ID1, inhibitor of DNA binding 1; HIF-1*α*, hypoxia-inducible factor-1 alpha; H/R, hypoxia-reoxygenation.

To study the function of ID1, siRNA was used to inhibit ID1 expression. The mRNA levels of ID1 were lowered significantly through the whole H/R process, and the protein levels were also reduced markedly (Figures 6(a) and 6(b)).

As ID1 was inhibited, the protein expression of HIF-1*α* was reduced after exposure to hypoxia for 24 hours (Figure 6(a)). The mRNA levels of HIF-1*α* were suppressed significantly at the end of the hypoxic treatment and the early stage of reoxygenation (Figure 6(c)). These results demonstrate that ID1 is critical for the induction of HIF-1*α* expression during H/R in HK-2 cells, and ID1 can regulate HIF-1*α* expression at the transcription level.

### 3.5. Twist Is Induced in the H/R Model and Is Regulated by HIF-1*α* and ID1

**Figure 7:**
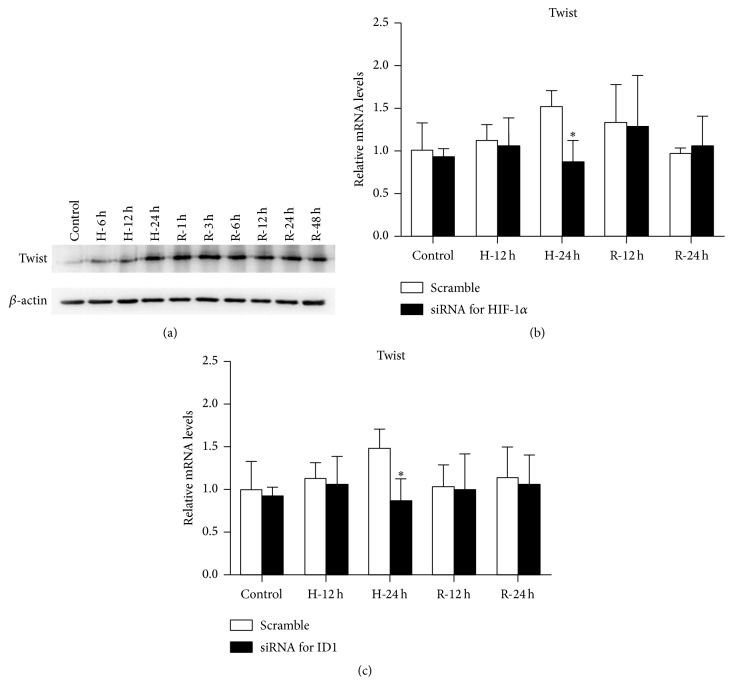
Twist is induced in the H/R model and is regulated by HIF-1*α* and ID1. (a) Western blot analysis of twist expression in HK-2 cells during H/R. *β*-actin was examined as the loading control. A representative blot from 3 independent experiments is shown. (b) In cells transfected with HIF-1*α* siRNA, and relative mRNA levels of twist were significantly reduced after 24 hours of hypoxia. *β*-actin mRNA served as the internal control (*n* = 3). ^*∗*^ *P* < .05 compared with the negative control (scramble). (c) In cells transfected with ID1 siRNA, relative mRNA levels of twist were significantly reduced after 24 hours of hypoxia. *β*-actin mRNA served as the internal control (*n* = 3). ^*∗*^ *P* < .05 compared with the negative control (scramble). ID1, inhibitor of DNA binding 1; HIF-1*α*, hypoxia-inducible factor-1 alpha; H/R, hypoxia-reoxygenation.

Twist is a bHLH transcription factor that can be induced by HIF-1*α* in TECs subjected to hypoxia [11]. In the present study, the expression of twist in the H/R model was observed. Twist was induced during hypoxia and reoxygenation at the protein level (Figure 7(a)).

To explore whether twist could be mediated by HIF-1*α*, the changes in twist when HIF-1*α* was silenced by siRNA transfection were measured (Figure 7(b)). Silencing HIF-1*α* could reduce the mRNA levels of twist in HK-2 cells during H/R, showing that HIF-1*α* may regulate the expression of twist at the transcriptional level. In addition, the mRNA levels of twist were decreased as ID1 was repressed endogenously (Figure 7(c)). Thus, twist seems to be regulated by both ID1 and HIF-1*α*.

## 4. Discussion

In this study, the authors observed the induction of ID1 in rat renal I/R injury and demonstrated that its expression was mainly localized in TECs. In the in vitro H/R model, ID1 was upregulated during both hypoxia and reoxygenation. The expression of HIF-1*α* was observed to be suppressed by siRNA and ID1 was found to be inhibited at the transcriptional level, which suggests that HIF-1*α* may regulate the induction of ID1 in the H/R model. In addition, according to previous findings, ID1 may have the ability to regulate HIF-1*α* [6]. Hence, the authors inhibited the expression of ID1 and demonstrated that ID1 could regulate HIF-1*α* at the transcriptional level and was critical for the induction of HIF-1*α* during H/R.

The renal I/R injury model is already a mature animal model that mimics clinical situations. In a previous study [10], the authors successfully established the renal I/R model that was applied in the present study. However, there is no in vitro I/R model that is generally accepted worldwide. Sauvant et al. set up an in vitro model of I/R, in which NRK-52E cells were exposed to hypoxia in a chamber with acid (pH 6.6) and glucose-deprived buffer for 2 hours and recultured under standard conditions for 48 hours [12]. Although most markers of apoptosis, dedifferentiation, and inflammation were expressed as anticipated, this model has not been widely applied, likely because of its poor standardization and repeatability. Different durations of hypoxia between 2 and 24 hours have been applied in different studies of AKI [13–15]. In their preliminary experiments, the authors observed the expression of HIF-1*α* after hypoxia for 6, 12, and 24 hours. With several repetitions, they found that only hypoxia exposure for 24 hours could induce stable upregulation of HIF-1*α* and vimentin.

In the H/R model, it was demonstrated that HIF-1*α* is induced during both hypoxia and reoxygenation and the dynamic expression of HIF-1*α* was shown following intensive observation. The preliminary study found that HIF-1*α* protein accumulates instantly after hypoxia for 1 hour. Conde et al. also identified the biphasic expression of HIF-1*α* in a HK-2 H/R model, but the duration of hypoxia was different from this study and they did not describe the expression process in detail [5]. In addition, this study confirmed that HIF-1*α* mRNA expression was significantly elevated during hypoxia and reoxygenation in HK-2 cells. Evidence has shown that hypoxia and oxidative stress can activate transcription of HIF-1*α* mRNA in other cell lines [16]. In contrast, no significant changes in HIF-1*α* mRNA expression were observed in the study by Conde et al., and this may mainly be attributed to a short hypoxia time and insufficient stimulation [5]. According to the results of this study, the authors consider that HIF-1*α* protein instantly accumulated at the start of exposure to hypoxia and its expression was induced following responses that relieve hypoxic injury and promote cellular repair. If exposure to hypoxia continues, transcription of HIF-1*α* may be elevated to ensure or increase its expression.

It has been reported that ID1 expression is induced exclusively in the degenerated, dilated renal tubular epithelium after unilateral ureteral obstruction [9]. The findings from the present study indicated that ID1 was significantly induced and mainly expressed in renal TECs during in vivo reperfusion after acute ischemia, which means ID1 may have important functions in AKI. Through an in vitro H/R model, the authors found that ID1 expression is markedly elevated during hypoxia at both the protein and mRNA levels. To determine whether HIF-1*α* is necessary for the induction of ID1 during hypoxia and reoxygenation, HIF-1*α* was silenced and a reduction of ID1 mRNA was observed. Therefore, HIF-1*α* could regulate ID1 transcriptionally as seen in HK-2 cells. In neuroblastoma cells [17], ID1 is downregulated in a hypoxic situation, but it is upregulated by HIF-1*α* in the absence of hypoxia-induced ATF-3. Therefore, it is obvious that the expression of ID1 under hypoxia differs in different cell lines, and the mechanism by which HIF-1*α* regulates ID1 may also be different and requires further research.

ID1 has been a hot topic in cancer research for years, and numerous cancer cells are reported to overexpress ID1, which is in relation to tumor angiogenesis. In renal TECs, ID1 was shown to drive dedifferentiation by suppressing E-cadherin expression [9]. Moreover, ID1 is involved in cell cycle progression and development and probably cross talks with HIF-1*α*. ID1 can stabilize HIF-1*α* protein in hepatocellular carcinoma cells [18]. In the study by Kim et al., ID1 was shown to enhance nuclear translocation and the transcriptional activity of HIF-1*α* by recruiting CBP into endothelial cells [6]. As there is potential for regulation of HIF-1*α* by ID1, this study explored the relationship between HIF-1*α* and ID1. The results demonstrated that ID1 is a regulator of HIF-1*α*. The induction of HIF-1*α* transcription was inhibited when ID1 was silenced. Thus, the authors hypothesized that HIF-1*α* could induce the increased expression of ID1 during H/R, and the augmented ID1 expression could elevate HIF-1*α* expression in return, which acts as a positive feedback loop.

Twist is an important member of the bHLH family and a master regulator of gastrulation and mesoderm specification that is also essential in mediating metastasis [19, 20]. Several studies have shown that twist may be regulated by HIF-1*α* and ID1. The transcriptional activity of twist is inhibited by ID1 in a kidney fibroblast cell line [21]. HIF-1*α* can induce twist expression in TECs subjected to hypoxia, leading to epithelial-to-mesenchymal transition [11]. The present study indicates that HIF-1*α* can regulate twist during H/R in HK-2 cells, and the transcription of twist could also be regulated by ID1. Thus, the mechanism of the regulation of twist expression by ID1 will be a focus of future research.

In summary, the results presented here demonstrate the induction of ID1 expression in renal TECs during in vivo and in vitro I/R injury, which could regulate the expression of HIF-1*α* transcriptionally. As a critical and protective factor for the repair of TECs, HIF-1*α* has been considered to be an important therapeutic target. Interventions based on its regulator, ID1, may offer new insight into strategies for ameliorating I/R injury.
